# Sarcopenia Severity Based on Computed Tomography Image Analysis in Patients with Cirrhosis

**DOI:** 10.3390/nu12113463

**Published:** 2020-11-11

**Authors:** Maryam Ebadi, Rahima A. Bhanji, Abha R. Dunichand-Hoedl, Vera C. Mazurak, Vickie E. Baracos, Aldo J. Montano-Loza

**Affiliations:** 1 Division of Gastroenterology & Liver Unit, University of Alberta, Edmonton, AB T6G 2X8, Canada; rbhanji@ualberta.ca; 2 Division of Human Nutrition, University of Alberta, Edmonton, AB T6G 2P5, Canada; abha@ualberta.ca (A.R.D.-H.); vmazurak@ualberta.ca (V.C.M.); 3 Department of Oncology, Cross Cancer Institute, Edmonton, AB T6G 1Z2, Canada; Vickie.Baracos@albertahealthservices.ca

**Keywords:** muscle loss, body composition, CT image

## Abstract

Standardized sex-specific cut-offs for sarcopenia in cirrhosis are needed to identify the risk of clinical complications and to discriminate the severity of sarcopenia. We aimed to compare clinical characteristics between patients with cirrhosis categorized according to the severity of sarcopenia. Computed tomography images were taken at the 3rd lumbar vertebra from 603 patients with cirrhosis and 129 adult donors for living liver transplantation. Patients with skeletal muscle index (SMI) two standard deviations (SD) below the sex-specific mean value of young donors (18–40 years old) were categorized as having severe sarcopenia whereas patients with SMI between −1 and −2 SD of the sex-specific young adult mean values were categorized as having sarcopenia. In the cirrhosis group, 408 patients (68%) were male with the mean age of 57 ± 0.4 years, and MELD score of 14 ± 0.4. Patients were divided into three groups: severe-sarcopenic (SMI < 30 cm^2^/m^2^ in females and <42 cm^2^/m^2^ in males), sarcopenic (30 ≤ SMI < 37 cm^2^/m^2^ in females and 42 ≤ SMI < 50 cm^2^/m^2^ in males) and non-sarcopenic (SMI ≥ 37 cm^2^/m^2^ in females and ≥50 cm^2^/m^2^ in males). Patients with cirrhosis and severe sarcopenia had lower muscle radiodensity and higher plasma neutrophil as well as neutrophil to lymphocyte ratio levels than both non- and sarcopenic groups. The frequency of alcohol-induced cirrhosis, refractory ascites, hepatic encephalopathy, CRP > 20 mg/mL, and severe malnutrition was also higher in severe-sarcopenic patients. The interval between sarcopenia and severe sarcopenia may reflect a window of opportunity in which to intervene and mitigate muscle wasting to improve patient outcomes.

## 1. Introduction

High-resolution image-based techniques such as computed tomography (CT) have expanded our understanding of the prognostic significance of body composition in patients with cirrhosis over the last decade. Secondary analysis of CT images is objective, precise, and is considered to be a gold standard to diagnose skeletal muscle abnormalities in clinical research. The most common muscle abnormality, sarcopenia (severe muscle depletion) associates with adverse outcomes in patients with cirrhosis [1]. Our group, and many others have shown sarcopenia to be a significant risk factor for overall mortality, waitlist mortality, higher frequency of infection, longer hospital stay, hepatic encephalopathy, poor quality of life, increased health care cost and post-liver transplantation (LT) death [2].

The prevalence of this radiologically-identified abnormality in skeletal muscle varies widely between 30–70%, in patients with cirrhosis [3]. This variability could result from divergent modalities applied to quantify muscle mass, criteria, cut-points used to define sarcopenia, and based on the population of interest. Therefore, standardized diagnostic criteria for sarcopenia in clinical research and practice are required to improve our ability to study this critical condition.

CT-measured skeletal muscle index (SMI, cm^2^/m^2^) at the level of the third lumbar vertebrate (L3) is a precise and effective indicator of whole-body muscle mass (R^2^ = 0.855, *p* < 0.01) [4]. Commonly, sarcopenia in cirrhosis has been defined as low muscle mass associated with adverse clinical outcomes. The recommended cut-offs of SMI < 50 cm^2^/m^2^ in male and < 39 cm^2^/m^2^ in female patients have been proposed for individuals awaiting LT at North American LT centers [5]. Compared to mortality related cut-offs, reference values derived from a young, healthy population may enable a valuable comparison of several clinical outcomes. Additionally, there is a need to reliably discriminate sarcopenia severity in patients with cirrhosis to implement early and effective clinical decisions. In this study, we aimed to define sarcopenia by degree of severity in patients with cirrhosis and to compare clinical characteristics between these groups.

## 2. Materials and Methods 

### 2.1. Study Population 

This retrospective, cross-sectional study was approved by the Institutional Review Board of the University of Alberta. All subjects gave their informed consent for inclusion before they participated in the study. The study was conducted in accordance with the Declaration of Helsinki, and the protocol was approved by the Ethics Committee (Pro00066572). Healthy controls and patients with cirrhosis who had a CT done as part of the routine LT assessment at the University of Alberta Hospital were included. Healthy controls (*n* = 129) were adult donors for living LT between the age of 19–58 who tested negative for viral hepatitis B and C serologies, without previous hepatic resections, or major abdominal surgeries. These individuals did not have a history of alcohol abuse, illicit drug use, and had no evidence of non-alcoholic fatty liver disease. 

The population of patients with cirrhosis for this analysis was derived from patients enrolled in the largest body composition analysis in North America with 677 patients [6]. The parent study was designed to investigate an association between body composition and survival. Of those 677 patients with cirrhosis, we were able to collect systemic physiological, metabolic parameters (albumin, WBC, platelets, neutrophils, lymphocytes, C-reactive protein (CRP), ferritin, iron, homocysteine, vitamin D) as well as nutritional status, assessed by subjective global assessment (SGA), for 603 patients within three months of the CT. Other clinical and demographic features of the patients were retrieved from the Alberta Liver Transplant database.

### 2.2. CT Image Analysis

Secondary analysis of abdominal CT scans taken at L3 was performed using Slice-O-Matic software (V4.2; Tomovision, Montreal, QC, Canada) to quantify body composition parameters. To estimate the cross-sectional area of each tissue, standard Hounsfield Unit (HU) thresholds of −29 to 150 HU for skeletal muscle, −150 to −50 HU for visceral adipose tissue [7] and −190 to −30 HU for subcutaneous adipose tissue [8] were applied. The total skeletal muscle visualized at L3 includes the psoas, erector spinae, quadratus lumborum, transversus abdominis, external and internal obliques, and rectus abdominis muscles. Using these HUs, a trained operator skilled in anatomy, demarked adipose tissue within the abdominal wall as visceral adipose tissue while the area between the skin line and outer abdominal wall was assessed for subcutaneous adipose tissue cross-sectional area estimation. The given tissue pixels were summed, multiplied by the pixel surface areas and consequently, tissue cross-sectional areas (cm^2^) were computed.

Tissue areas (cm^2^) were normalized to the patient height squared (cm^2^/m^2^) and reported as indexes in cm^2^/m^2^ to quantify three body composition variables including visceral adipose tissue index (VATI), subcutaneous adipose tissue index (SATI), and skeletal muscle index (SMI). Muscle radiodensity estimation, mean muscle radiation tissue attenuation (HU), was estimated for the entire cross-sectional area at L3, which correlates with the triglyceride content of muscle [9].

### 2.3. Statistical Analysis

Continuous variables are described as mean and standard deviation (SD) and differences in means were compared using independent *t*-test. Categorical variables are presented as percentages and associations between these variables were determined using Fisher’s exact test. Correlation between SMI with age and BMI was determined by Pearson’s correlation coefficient (*r*) analysis. One-way ANOVA with Bonferroni posthoc comparisons was used to compare clinical characteristics and metabolic profiles between sarcopenic groups. For comparisons of medians in box-plot, the Kruskal-Wallis test was used. Overall survival estimates were obtained using Kaplan-Meier curves and comparisons between groups were conducted using the log-rank test.

Severe sarcopenia was defined as SMI two SD below the sex-specific mean value of a young donor (18–40 years old) whereas sarcopenia was defined as SMI between −1 and −2 SD of sex-specific young donor mean values. The non-sarcopenic group included patients with SMI higher than one SD below the sex-specific mean for young donors. Statistical analyses were performed using SPSS (SPSS for Windows, version 26.0, SPSS, Chicago, IL, USA) and a difference was considered to be statistically significant if the *p*-value was less than 0.05.

## 3. Results

### 3.1. Baseline Characteristics 

Among donors, 55 were male (43%) with a mean age of 35 ± 1 years (Table 1). In the cirrhosis group, 408 patients (68%) were male with a mean age of 57 ± 0.4 years, and MELD score of 14 ± 0.4. The most common reason for cirrhosis was hepatitis C (40%), followed by NASH (23%), alcohol (22%), autoimmune liver disease (8%), and hepatitis B (6%). Decompensation with ascites and hepatic encephalopathy was present in 28% and 32% of patients, respectively.

### 3.2. Body Composition Analysis in Donors and Patients with Cirrhosis

BMI was significantly higher in male donors compared to females (26 ± 0.5 vs. 25 ± 0.4; *p* = 0.009); there was no difference in BMI by sex in patients with cirrhosis. In both donors and patients with cirrhosis, body composition differed between males and females, with male patients having greater SMI and VATI whereas SATI was higher in females (*p* ≤ 0.001 for each; Table 1). There was no difference in muscle radiodensity between male and female donors; muscle radiodensity was significantly lower in female patients with cirrhosis compared to their male counterparts (32 ± 0.6 vs. 37 ± 0.4 HU; *p* < 0.001). 

**Figure 1:**
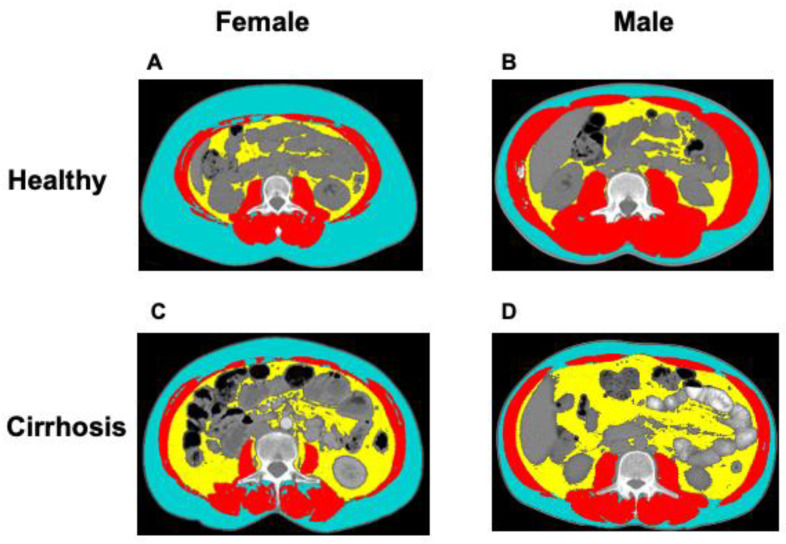
Abdominal computed tomography (CT) images taken at the 3rd. lumbar vertebra of healthy donors and patients with cirrhosis applied for body composition assessment. Example of body composition features in a (**A**) female healthy donor, (**B**) male healthy donor (**C**) female patient with cirrhosis, and (**D**) male patient with cirrhosis. Muscle, subcutaneous, and visceral adipose tissue are shown in red, blue, and yellow, respectively.

With regard to the features of body composition, female patients with cirrhosis had significantly higher BMI (27 ± 0.5 vs. 25 ± 0.4 kg/m^2^; *p* < 0.001) and VATI (32 ± 2 vs. 14 ± 1 cm^2^/m^2^; *p* < 0.001), but significantly lower muscle radiodensity (32 ± 0.6 vs. 43 ± 0.6 HU; *p* < 0.001) when compared to female donors. Male patients with cirrhosis had higher BMI (28 ± 0.3 vs. 26 ± 0.5 kg/m^2^; *p* = 0.04), and VATI (40 ± 1 vs. 32 ± 3 cm^2^/m^2^; *p* = 0.08), but lower SMI (53 ± 0.4 vs. 57 ± 1 cm^2^/m^2^; *p* = 0.002), and muscle radiodensity (37 ± 0.4 vs. 44 ± 0.7 HU; *p* < 0.001) compared to donors. An example of a CT image taken at the 3rd. lumbar vertebra of healthy donors and patients with cirrhosis, with similar BMI, is presented in Figure 1.

### 3.3. Sex-Specific Cut-Off Values for Sarcopenia 

**Figure 2:**
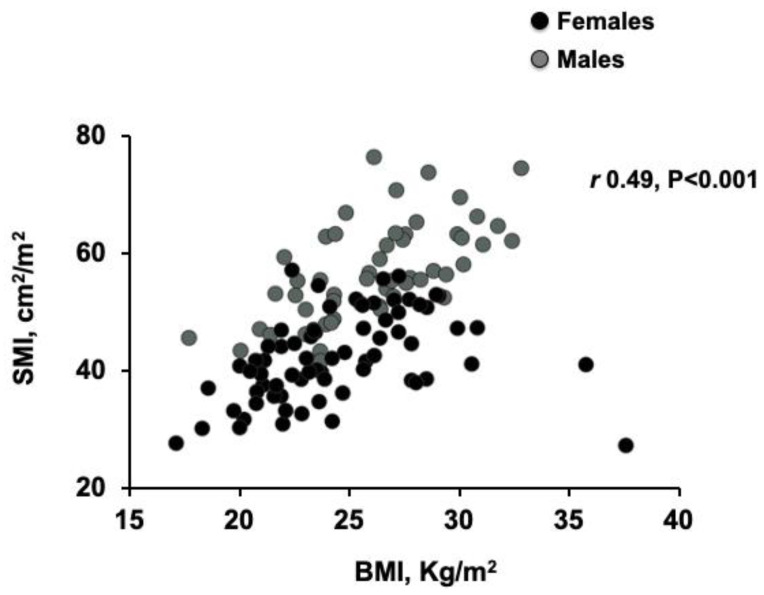
Scatter graph depicting correlations between skeletal muscle index (SMI) and body mass index (BMI) in donors. Moderate correlation (Pearson’s correlation) between SMI and BMI in donors (*r* = 0.49, *p* < 0.001).

**Figure 3:**
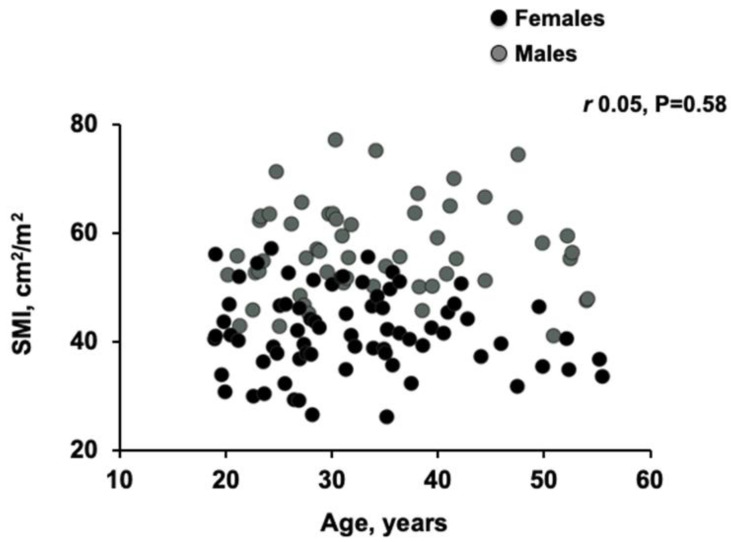
Scatter graph depicting correlations between skeletal muscle index (SMI) and age in donors. There was not any significant linear correlation (Pearson’s correlation) between SMI and age in donors (*r* = 0.05, *p* = 0.58).

There was a moderate linear correlation between BMI and SMI in donors (*r* = 0.49, *p* < 0.001; Figure 2), which was stronger in patients with BMI < 35 kg/m^2^ (*r* = 0.63, *p* < 0.001). The modest increase in SMI observed in patients with a BMI between 20 to 35 was not observed with increasing age (*r* = 0.05, *p* = 0.58; Figure 3).

Cut-offs for severe sarcopenia were SMI two SD below the young healthy donor mean and were <42 cm^2^/m^2^ for men and <30 cm^2^/m^2^ for women. Cut-offs for sarcopenia were SMI values between −1 and −2 SD of the sex-specific mean for young adults and were <50 cm^2^/m^2^ in men and <37 cm^2^/m^2^ in women.

Using these values, patients were divided into three groups: severe-sarcopenic (SMI < 30 cm^2^/m^2^ in females and <42 cm^2^/m^2^ in males), sarcopenic (30 ≤ SMI < 37 cm^2^/m^2^ in females and 42 ≤ SMI < 50 cm^2^/m^2^ in males) and non-sarcopenic (SMI ≥ 37 cm^2^/m^2^ in females and ≥50 cm^2^/m^2^ in males), Table 2. Fifty patients (8%) were in the severe-sarcopenic group, 138 (23%) in the sarcopenic group and 415 (69%) patients were in the non-sarcopenic group. Forty-one out of 50 (82%) patients with severe-sarcopenia and 112 out of 138 (81%) sarcopenic patients were male.

### 3.4. Clinical Features of Patients with Cirrhosis and Sarcopenia 

Severely-sarcopenic patients were mostly males with alcoholic cirrhosis (Table 3). Not surprisingly, the frequency of hepatic encephalopathy was significantly higher in this group compared to those in the sarcopenic and non-sarcopenic groups (52% vs. 38% and 28%, *p* < 0.001 for each). Although the mean CRP values did not differ between groups, a higher frequency of patients with CRP ≥ 20 mg/L [10] was seen in patients with severe sarcopenia. Patients with cirrhosis and severe sarcopenia had plasma neutrophil, homocysteine, as well as neutrophil to lymphocyte ratio levels higher than both non- and sarcopenic groups (Table 3).

The prevalence of patients with severe malnutrition, determined as SGA category C was significantly higher in patients with severe sarcopenia compared to both, sarcopenic and non-sarcopenic groups (33% vs. 8% and 8%, *p* = 0.001 for each). 

Patients in the severely sarcopenic group had a lower BMI (23 ± 0.9 vs. 29 ± 0.3, *p* < 0.001), SATI (30 ± 3 vs. 64 ± 2, *p* < 0.001) and VATI (25 ± 2 vs. 41 ± 1, *p* < 0.001) compared to non-sarcopenic patients.

**Figure 4:**
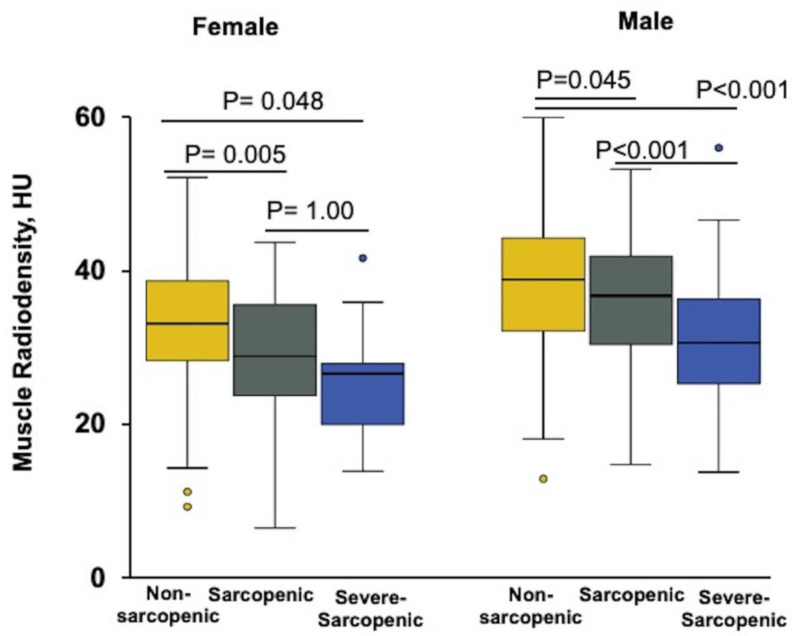
Box and whisker plot of the muscle radiodensity for male and female patients stratified according to sarcopenia severity. Among female patients, there was no significant difference in the median muscle radiodensity (HU) between severe-sarcopenic and sarcopenic patients. In contrast, male patients with cirrhosis and severe sarcopenia had significantly lower muscle radiodensity compared to both non-sarcopenic (*p* = 0.045) and sarcopenic patients (*p* < 0.001).

There was no difference in BMI, SATI, and VATI between the sarcopenic and severe-sarcopenic patients, but muscle radiodensity was significantly lower in severe-sarcopenic group (29 ± 1HU) compared to sarcopenic (34 ± 0.8, *p* = 0.001) and non-sarcopenic groups (36 ± 0.4HU, *p* < 0.001). When stratified by sex, muscle radiodensity was significantly lower in severe-sarcopenic than sarcopenic patients, but only in males (*p* < 0.001); this association was not seen in female patients (Figure 4). No significant difference was observed between the three groups with regard to age, ascites, vitamin D deficiency, and plasma levels of albumin, WBC as well as lymphocytes.

### 3.5. Survival of Patients with Cirrhosis and Sarcopenia 

**Figure 5:**
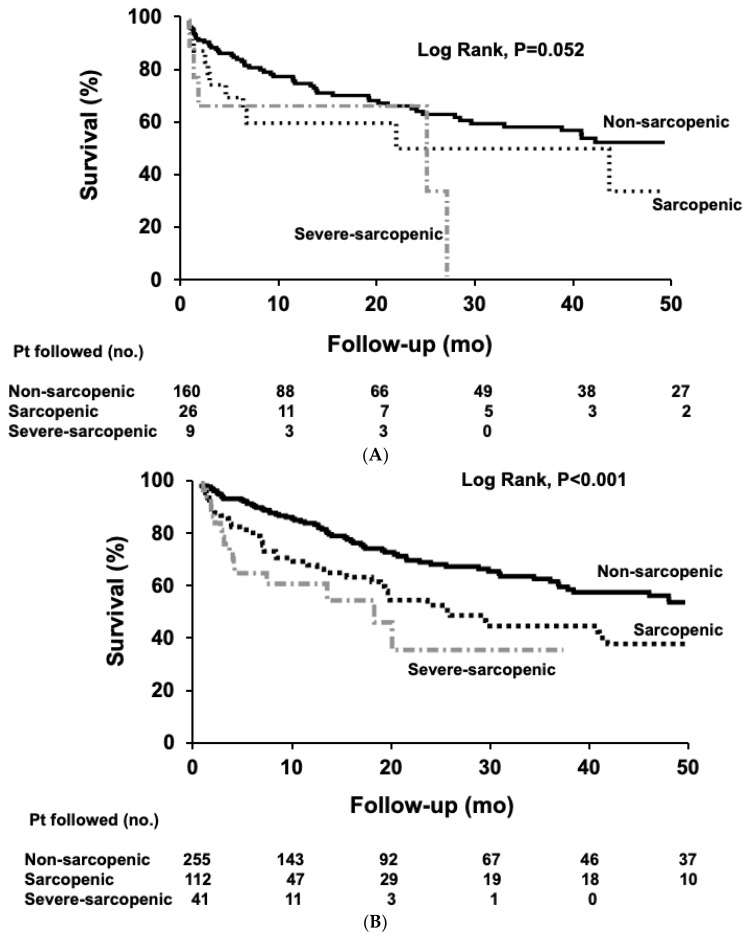
Survival curves in patients with cirrhosis according to the sarcopenia severity. (**A**) Females (**B**) Males. Survival over time was assessed using Kaplan-Meier curves and the curves were compared using the log-rank test. Significantly longer median survival was observed in non-sarcopenic patients compared to the sarcopenic and severe-sarcopenic patients, in males. (Log-rank < 0.001). Pt, patient; mo, months; no, number.

Patients were followed for a median time of 41 months, until death (*n* = 219), LT (*n* = 223), or censoring (*n* = 161). Overall median survival was shorter in patients with severe sarcopenia (20 months; 95% CI, 9–30) and sarcopenia (25 months; 95% CI, 15–34) than in patients without sarcopenia (62 months; 95% CI, 26–98; *p* < 0.001; log-rank test). In females, median survival tended to be shorter in patients with severe sarcopenia (25 months; 95% CI, 0.1–60) and sarcopenia (22 months; 95% CI, 0.1–57) compared to non-sarcopenic patients (58 months; 95% CI, 24–93; *p* = 0.052; log-rank test, Figure 5A). Median survival was significantly longer in non-sarcopenic male patients (62 months; 95% CI, 16–108) compared to sarcopenic (25 months; 95% CI, 15–35) and severe-sarcopenic male patients (18 months; 95% CI, 2–34; *p* < 0.001; log-rank test, Figure 5B).

## 4. Discussion

This study is the first to evaluate cut-offs for sarcopenia based on a reference young, healthy population. We investigated body composition differences by sex between patients with liver cirrhosis and healthy donors. Standardizing cut-off values for sarcopenia in cirrhosis will limit the heterogeneity seen in sarcopenia literature and will allow for evaluation of muscle abnormalities in clinical practice. Using these cut-offs may allow for early identification of patients with muscle abnormality, providing a window of opportunity in which to intervene, whereby improving clinical outcomes. We show the frequency of complications, inflammation, poor nutritional status, and abnormal body composition features to be significantly higher in patients with severe sarcopenia when compared to those in the sarcopenic and non-sarcopenic groups.

We also evaluated inflammatory biomarkers of sarcopenia including homocysteine, CRP, NLR and vitamin D. Severe vitamin D deficiency was similar in all groups. We showed levels of homocysteine, CRP and NLR were higher in patients with severe sarcopenia, suggesting a major role of inflammation in sarcopenia severity in cirrhosis. The association of systemic inflammation with sarcopenia severity has not been clearly described in cirrhosis. However, the role of inflammation in sarcopenia progression has been suggested in various studies, as reviewed by Meng et al. [11]. Homocysteine associates with several pathways involved in sarcopenia such as oxidative stress, inflammatory myopathies, and fibrosis [12]. The NLR is a practical indicator of systemic inflammation which was associated with higher risk of sarcopenia in cancer patients [13]. It would be intriguing to study the beneficial impact of anti-inflammatory agents such as long chain n-3 fatty acids in reducing the severity of muscle loss in future clinical trials.

Not surprisingly, cirrhosis complications such as refractory ascites and hepatic encephalopathy were more common in patients with severe sarcopenia. This may be explained by decreased food intake and early satiety, due to ascites [14], leading to exhaustion of muscle protein [2] and severe depletion of muscle protein stores in cirrhosis. Chronic protein-energy malnutrition can be assessed by SGA in patients with cirrhosis [15]. Nutritional interventions with branched chain amino acids may help to improve nutritional status and related complications in cirrhosis [2].

Whereas CT-based diagnosis of sarcopenia is objective and use of standardized cut-offs may allow for risk stratification there are certain limitations. For example, radiation exposure may be an additional barrier in recruiting healthy populations to determine appropriate cut-offs in various reference populations. Trauma patients have frequently been used as a reference population in body composition studies [16,17], but this practice remains questionable. Living donors represent the healthy, normal population and serve as an appropriate reference for body composition assessment.

In this study, two distinct classes of sarcopenia severity were established for use in clinical practice. Comparison between sarcopenia cut-off values established based on reference values and mortality related cut-offs in cirrhosis [5] suggest even a small amount of muscle loss to be clinically significant and to be related to mortality in cirrhosis. Cut-off values for SMI derived from young healthy donors in this study were SMI < 42 cm^2^/m^2^ in men and <30 cm^2^/m^2^ in women; these values are in accordance with cut-offs recently established in a healthy Caucasian population (SMI below 43.5 cm^2^/m^2^ for males and 30 cm^2^/m^2^ for females) [18]. Application of these values enables inclusion of patients who are at risk for adverse complications. Some of these patients may have the most benefit from intervention as they still have muscle mass that can be maintained. The validity of these CT-measured cut-off values should be confirmed in larger, future studies.

Fortunately, only 8% of patients with cirrhosis were in the severe-sarcopenic group in our study; majority of them had alcohol-related cirrhosis. This is in line with a previous study showing sarcopenia prevalence of 12.8% in patients with alcoholic cirrhosis [19] using the healthy values of body composition parameters. Patients with alcohol-related cirrhosis are at a higher risk for sarcopenia [20] due to increased muscle autophagy [21], impaired skeletal muscle protein synthesis [22] as well as the synergistic interaction of ethanol and hyperammonemia worsening muscle breakdown [21]. This small subset of patients with altered metabolic and inflammatory profiles as well as poor nutritional status presented with deteriorated liver function. Further investigation is needed on whether patients with severe sarcopenia should be considered for expedited LT to improve outcomes or be de-listed as a result of being too sick for LT.

SATI and VATI were also lower in sarcopenic patients, when compared to the non-sarcopenic group; this suggests there is increased fat utilization during conditions of high-energy demand prior to onset of severe-sarcopenia development. This metabolic flexibility may help to preserve muscle prior to progression seen in advanced stages of liver disease. Nutrition and physical activity interventions remain the cornerstone of treatment for preventing and/or treating sarcopenia. Oncological research has shown interventions designed to attenuate muscle wasting should be applied earlier when anabolic capacity is preserved [23]. We feel patients in the sarcopenic group may be in this stage in comparison to those who are in the severe-sarcopenic group. Thus, interventions are more likely to be effective in the former group.

Although muscle radiodensity did not differ between male and female donors, it was lower in patients with cirrhosis suggesting alteration in muscle quality with accumulation of fat in patients with cirrhosis. At the early stages of muscle loss (sarcopenia), female muscle is infiltrated with higher amounts of fat as evidenced by lower muscle radiodensity. Female muscle has a higher proportion of oxidative fibers and the capability to accumulate more fat than men [2]. This metabolic flexibility in female patients with cirrhosis to store and oxidize more fat may explain the lower prevalence of sarcopenia seen in females; this finding needs further investigation.

We acknowledge there are limitations in this study. Due to the retrospective nature of the study, we were unable to evaluate systemic physiological and metabolic markers such as anabolic hormones (i.e., testosterone, estrogen, insulin, and insulin-like growth factor-I (IGF-I)) as well as physical function. We were unable to match patients with cirrhosis to donors by age due to the small number of patients over 50 years old (*n* = 13). Though there was lack of association between age and SMI in our healthy donors (*r* = 0.05, *p* = 0.58) and no substantial decline in SMI in those over the age of 50 years was seen in previous studies [18]. The small number of donors in this study also limits the generalizability of healthy cut-offs; Larger studies are required to validate established cut-offs as normative reference data. BMI-dependent cut-off values and the impact of ethnicity should be investigated in future studies with larger number of reference populations.

## 5. Conclusions

In conclusion, cirrhosis is associated with substantial changes in body composition when compared to healthy controls. When cut-offs derived from CT scans of healthy living donors were utilized to discriminate the severity of CT-measured sarcopenia, the frequency of complications, inflammation, poor nutritional status, and abnormal body composition features were significantly higher in patients with severe sarcopenia compared to sarcopenic and non-sarcopenic patients. When using a single CT, the stage between a normal SMI and sarcopenia may be the perfect window of opportunity in which to intervene. This may mitigate sarcopenia whereby improving patient outcomes. However, loss of SMI on follow-up imaging may suggest requirement for a more aggressive intervention.

## Figures and Tables

**Table 1:** Body composition features of healthy donors and patients with cirrhosis by sex.

	Donor			Cirrhosis			*p*-Value Female (Donor vs. Cirrhosis)	*p*-Value Male (Donor vs. Cirrhosis)
	Female (*n* = 74)	Male (*n* = 55)	*p*-Value	Female (*n* = 195)	Male (*n* = 408)	*p*-Value		
Age, years	33 ± 1	35 ± 1	0.20	56 ± 0.7	57 ± 0.4	0.17	<0.001	<0.001
BMI, (kg/m^2^)	25 ± 0.4	26 ± 0.5	0.009	27 ± 0.5	28 ± 0.3	0.95	<0.001	0.04
SMI, (cm^2^/m^2^)	44 ± 0.8	57 ± 1	<0.001	45 ± 0.7	53 ± 0.4	<0.001	0.27	0.002
SATI, (cm^2^/m^2^)	65 ± 4	47 ± 3	0.001	68 ± 4	49 ± 2	<0.001	0.60	0.57
VATI, (cm^2^/m^2^)	14 ± 1	32 ± 3	<0.001	32 ± 2	40 ± 1	0.001	<0.001	0.08
Muscle radiodensity, HU	43 ± 0.6	44 ± 0.7	0.37	32 ± 0.6	37 ± 0.4	<0.001	<0.001	<0.001

Abbreviations: BMI, Body mass index; HU, Hounsfield unit; SATI, Subcutaneous adipose index; SMI, Skeletal muscle index; VATI, Visceral adipose tissue index.

**Table 2:** Sex-specific cut-off values of the skeletal muscle index (cm^2^/m^2^) for sarcopenia in cirrhosis.

	Female	Male
Skeletal muscle index two SD below the mean of a young healthy donor	30	42
Skeletal muscle index one SD below the mean of a young healthy donor	37	50
Severe-Sarcopenic	<30	<42
Sarcopenic	30 ≤ SMI < 37	45 ≤ SMI < 50
Non-sarcopenic	≥37	≥50

SD: standard deviation; SMI: skeletal muscle index.

**Table 3:** Clinical characteristics and metabolic profile of patients with cirrhosis and different severity of sarcopenia.

	Non-Sarcopenic (*n* = 415)	Sarcopenic (*n* = 138)	Severe-Sarcopenic (*n* = 50)	*p*-Value
Age, years	57 ± 0.4	56 ± 0.8	58 ± 1	0.37
Sex, male	255 (61)	112 (81)	41 (82)	<0.001
Cirrhosis ethology				
NASH	98 (24)	33 (24)	5 (10)	0.09
Alcohol	73 (18)	40 (29)	22 (44)	<0.001
Hepatitis C	180 (43)	48 (35)	15 (30)	0.06
Hepatitis B	27 (7)	7 (5)	4 (8)	0.73
Autoimmune liver diseases	34 (8)	10 (7)	3 (6)	0.83
Hepatic encephalopathy	116 (28)	52 (38)	26 (52)	0.001
Refractory ascites	88 (21)	49 (36)	21 (42)	<0.001
MELD score	14 ± 0.4	15 ± 0.7	16 ± 1	0.12
Severe-malnutrition (SGA C)	15 (8)	6 (8)	10 (33)	0.001
CRP> 20, mg/mL	54 (19)	19 (22)	16 (42)	0.01
Vitamin D deficiency (<50 nmol/L)	204 (57)	66 (59)	32 (73)	0.14
Albumin, g/L	33 ± 0.3	32 ± 0.6	33 ± 0.9	0.24
WBC, 10^9^/L	5.7 ± 0.2	5.9 ± 0.3	6.7 ± 0.5	0.23
Neutrophil count, 10^9^/L	3.7 ± 0.1	3.9 ± 0.2	5.2 ± 0.5 *^,¥^	0.001
Lymphocyte count, 10^9^/L	1.2 ± 0.04	1.1 ± 0.1	0.9 ± 0.06	0.09
Platelet, 10^9^/L	102 ± 3	124 ± 8 *	116 ± 7	0.005
Homocysteine, µmol/L	13 ± 0.4	14 ± 1	18 ± 2 *	0.007
CRP, mg/mL	16 ± 2	16 ± 3	28 ± 8	0.13
BMI, kg/m^2^	29 ± 0.3	25 ± 0.4 *	23 ± 0.9 *	<0.001
SATI, cm^2^/m^2^	64 ± 2	37 ± 2 *	30 ± 3 *	<0.001
VATI, cm^2^/m^2^	41 ± 1	30 ± 2 *	25 ± 2 *	<0.001
Muscle radiodensity, HU	36 ± 0.4	34 ± 0.8 *	29 ± 1 *^,¥^	<0.001
Neutrophil-lymphocyte ratio	4.5 ± 0.3	4.7 ± 0.3	7.5 ± 1 *^,¥^	0.001

* different than non-sarcopenic. ^¥^ different than sarcopenic. The subjective global assessment was performed in 290 patients. Abbreviations: BMI, body mass index; CRP, C-reactive protein; HU, Hounsfield unit; MELD, model for end-stage liver disease; NASH, non-alcoholic steatohepatitis; SATI, subcutaneous adipose index; SGA, subjective global assessment; VATI, visceral adipose tissue index; WBC, white blood cell.
